# Supplementary material for: Parallel evolution of urban–rural clines in melanism in a widespread mammal
Source: Sci Rep. 2022 Feb 2;12:1752. doi: 10.1038/s41598-022-05746-2 (PMC8810909; doi:10.1038/s41598-022-05746-2)
Supplement: Supplementary file 1 — Supplementary Information. [file 41598_2022_5746_MOESM1_ESM.pdf]

Supplementary Information

Parallel evolution of urban-rural clines in melanism in a widespread mammal

Bradley J. Cosentino[1] and James P. Gibbs[2]

[1]Department of Biology, Hobart and William Smith Colleges, 300 Pulteney Street, Geneva, NY 14886, USA

[2]Department of Environmental Biology, State University of New York College of Environmental Science and Forestry, 1 Forestry Drive, Syracuse, NY 13210, USA

This file includes:

Supplementary Table 1: Number of *Sciurus carolinensis* observations in total and by color morph in each city selected for analysis.

Supplementary Table 2: Parameter estimates for fixed effects in a linear mixed model of the probability of melanism in eastern gray squirrels (*Sciurus carolinensis*).

Supplementary Table 3: Parameter estimates for the random effect of city in a linear mixed model of the probability of melanism in eastern gray squirrels (*Sciurus carolinensis*). The estimated standard deviation for the random effect among cities was 1.68.

Supplementary Table 4: Parameter estimates for fixed effects in a linear mixed model of the probability of melanism in eastern gray squirrels (*Sciurus carolinensis*) using alternative spatial thinning distances (10 m, 50 m, 100 m).

Supplementary Table 5: Parameter estimates for fixed effects in a linear mixed model of the probability of melanism in eastern gray squirrels (*Sciurus carolinensis*) using alternative buffer distances to quantify impervious cover around each squirrel observation (500 m, 1 km, 10 km).

Supplementary Figure 1. Map of 43 cities in North America included in analyses of the distribution of coat color morphs of eastern gray squirrels (*Sciurus carolinensis*).

Supplementary Figure 2. Map of Syracuse, NY highlighting methodology for defining the spatial extent of the city footprint and buffer.

Supplementary Figure 3. Spatial correlograms of residuals from the linear mixed model of the probability of melanism. Moran's *I* is shown for distance classes of 1 km for a model either without or with a spatial autocovariate included in the model.

Supplementary Table 1. Number of *Sciurus carolinensis* observations in total and by color morph in each city selected for analysis.

City name	*S. carolinensis* observations	Gray morph	Melanic morph	Other morphs
Asheville, NC	164	142	0	22
Atlanta, GA	509	506	0	3
Baton Rouge, LA	177	171	3	3
Birmingham, AL	106	105	0	1
Boston, MA	3104	2971	84	49
Bowling Green, KY	164	108	0	56
Buffalo, NY	203	114	85	4
Burlington, VT	197	196	0	1
Cape Coral, FL	107	107	0	0
Charleston, SC	129	129	0	0
Charlotte, NC	237	233	3	1
Chicago, IL	1047	962	84	1
Cincinnati, OH	732	668	50	14
Cleveland, OH	298	183	111	4
Columbus, OH	332	281	17	34
Detroit, MI	282	116	159	7
Gainesville, FL	254	254	0	0
Geneva, NY	117	113	3	1
Houston, TX	885	878	4	3
Huntsville, AL	178	177	0	1
Kansas City, MO	101	98	0	3
Kitchener, Ontario	200	114	85	1
Miami, FL	777	774	0	3
Milwaukee, WI	346	328	11	7
Minneapolis, MN	570	426	40	104
Montréal, Quebec	770	648	80	42
Nashville, TN	117	116	0	1
New Orleans, LA	118	117	1	0
New York, NY	4731	4236	480	15
Orlando, FL	459	451	1	7
Ottawa, Ontario	697	230	466	1
Pittsburgh, PA	226	193	30	3
Raleigh, NC	681	668	1	12
Richmond, VA	267	258	1	8
Rochester, NY	122	113	6	3
Springfield, MA	140	117	23	0
St. Louis, MO	253	250	1	2
State College, PA	253	253	0	0
Syracuse, NY	736	546	189	1
Tampa, FL	1520	1518	1	1
Toronto, Ontario	1723	615	1075	33
Virginia Beach, VA	251	249	1	1
Washington DC	2644	2271	298	75

Supplementary Table 2. Parameter estimates for fixed effects in a linear mixed model of the probability of melanism in eastern gray squirrels (*Sciurus carolinensis*).

Fixed effect	Estimate	SE	*z*	*P*
Intercept	-3.73	0.35	-10.67	<0.001
Impervious cover	0.17	0.04	3.76	<0.001
Winter temperature	-2.14	0.33	-6.43	<0.001
Forest cover	-0.08	0.27	-0.30	0.766
City size	0.58	0.28	2.09	0.037
Impervious*Temperature	-0.07	0.07	-0.91	0.363
Impervious*Forest cover	0.15	0.03	4.92	<0.001
Impervious*City size	0.13	0.03	3.91	<0.001
Spatial autocovariate	0.72	0.03	22.28	<0.001

Supplementary Table 3. Parameter estimates for the random effect of city in a linear mixed model of the probability of melanism in eastern gray squirrels (*Sciurus carolinensis*). The estimated standard deviation for the random effect among cities was 1.68.

City name	Conditional modes
Asheville, NC	-1.20
Atlanta, GA	-2.17
Baton Rouge, LA	3.19
Birmingham, AL	-0.57
Boston, MA	-0.98
Bowling Green, KY	-0.62
Buffalo, NY	2.34
Burlington, VT	-2.98
Cape Coral, FL	-0.05
Charleston, SC	-0.38
Charlotte, NC	0.85
Chicago, IL	-1.14
Cincinnati, OH	0.79
Cleveland, OH	2.60
Columbus, OH	0.09
Detroit, MI	2.57
Gainesville, FL	-0.14
Geneva, NY	1.25
Houston, TX	1.05
Huntsville, AL	-1.00
Kansas City, MO	-2.33
Kitchener, Ontario	2.91
Miami, FL	-0.33
Milwaukee, WI	-1.72
Minneapolis, MN	-2.68
Montréal, Quebec	-1.38
Nashville, TN	-1.34
New Orleans, LA	2.16
New York, NY	0.24
Orlando, FL	1.58
Ottawa, Ontario	2.06
Pittsburgh, PA	1.14
Raleigh, NC	-1.16
Richmond, VA	-0.55
Rochester, NY	-0.23
Springfield, MA	1.94
St. Louis, MO	-2.19
State College, PA	-1.62
Syracuse, NY	2.37
Tampa, FL	0.59
Toronto, Ontario	2.77
Virginia Beach, VA	-0.37
Washington DC	2.22

Supplementary Table 4. Parameter estimates for fixed effects in a linear mixed model of the probability of melanism in eastern gray squirrels (*Sciurus carolinensis*) using alternative spatial thinning distances (10 m, 50 m, 100 m). Cities included in each dataset had a minimum of 100 squirrel observations. Samples sizes are included for the number of cities and squirrel observations included in each dataset.

Thinning distance	Cities	Squirrels	Fixed effect	Estimate	SE	*z*	*P*
10 m	43	26,924	Intercept	-3.73	0.35	-10.67	<0.001
			Impervious cover	0.17	0.04	3.76	<0.001
			Winter temperature	-2.14	0.33	-6.43	<0.001
			Forest cover	-0.08	0.27	-0.30	0.766
			City size	0.58	0.28	2.09	0.037
			Impervious*Temperature	-0.07	0.07	-0.91	0.363
			Impervious*Forest cover	0.15	0.03	4.92	<0.001
			Impervious*City size	0.13	0.03	3.91	<0.001
			Spatial autocovariate	0.72	0.03	22.28	<0.001
50 m	38	20,285	Intercept	-3.54	0.35	-10.13	<0.001
			Impervious cover	0.17	0.05	3.47	<0.001
			Winter temperature	-2.27	0.35	-6.45	<0.001
			Forest cover	-0.15	0.29	-0.52	0.600
			City size	0.60	0.32	1.91	0.056
			Impervious*Temperature	-0.04	0.08	-0.45	0.655
			Impervious*Forest cover	0.20	0.03	6.29	<0.001
			Impervious*City size	0.13	0.03	3.91	<0.001
			Spatial autocovariate	0.57	0.03	18.98	<0.001
100 m	34	16,991	Intercept	-3.43	0.34	-10.02	<0.001
			Impervious cover	0.18	0.05	3.50	<0.001
			Winter temperature	-2.07	0.33	-6.20	<0.001
			Forest cover	-0.07	0.27	-0.26	0.797
			City size	0.30	0.30	1.00	0.318
			Impervious*Temperature	-0.05	0.08	-0.54	0.592
			Impervious*Forest cover	0.22	0.03	6.46	<0.001
			Impervious*City size	0.13	0.03	3.86	<0.001
			Spatial autocovariate	0.49	0.03	16.64	<0.001

Supplementary Table 5. Parameter estimates for fixed effects in a linear mixed model of the probability of melanism in eastern gray squirrels (*Sciurus carolinensis*) using alternative buffer distances to quantify impervious cover around each squirrel observation (500 m, 1 km, 10 km).

Impervious cover buffer distance	Fixed effect	Estimate	SE	*z*	*P*
500 m	Intercept	-3.74	0.35	-10.71	<0.001
	Impervious cover	0.15	0.04	3.48	<0.001
	Winter temperature	-2.15	0.33	-6.45	<0.001
	Forest cover	-0.09	0.27	-0.32	0.748
	City size	0.60	0.28	2.17	0.030
	Impervious*Temperature	0.02	0.07	0.23	0.820
	Impervious*Forest cover	0.12	0.03	4.14	<0.001
	Impervious*City size	0.08	0.03	2.36	0.018
	Spatial autocovariate	0.70	0.03	21.97	<0.001
1 km	Intercept	-3.73	0.35	-10.67	<0.001
	Impervious cover	0.17	0.04	3.76	<0.001
	Winter temperature	-2.14	0.33	-6.43	<0.001
	Forest cover	-0.08	0.27	-0.30	0.766
	City size	0.58	0.28	2.09	0.037
	Impervious*Temperature	-0.07	0.07	-0.91	0.363
	Impervious*Forest cover	0.15	0.03	4.92	<0.001
	Impervious*City size	0.13	0.03	3.91	<0.001
	Spatial autocovariate	0.72	0.03	22.28	<0.001
10 km	Intercept	-3.80	0.36	-10.68	<0.001
	Impervious cover	0.26	0.05	5.41	<0.001
	Winter temperature	-2.15	0.34	-6.35	<0.001
	Forest cover	-0.10	0.27	-0.38	0.708
	City size	0.58	0.28	2.06	0.040
	Impervious*Temperature	-0.06	0.08	-0.81	0.421
	Impervious*Forest cover	0.23	0.03	7.29	<0.001
	Impervious*City size	0.23	0.04	6.27	<0.001
	Spatial autocovariate	0.74	0.03	23.25	<0.001

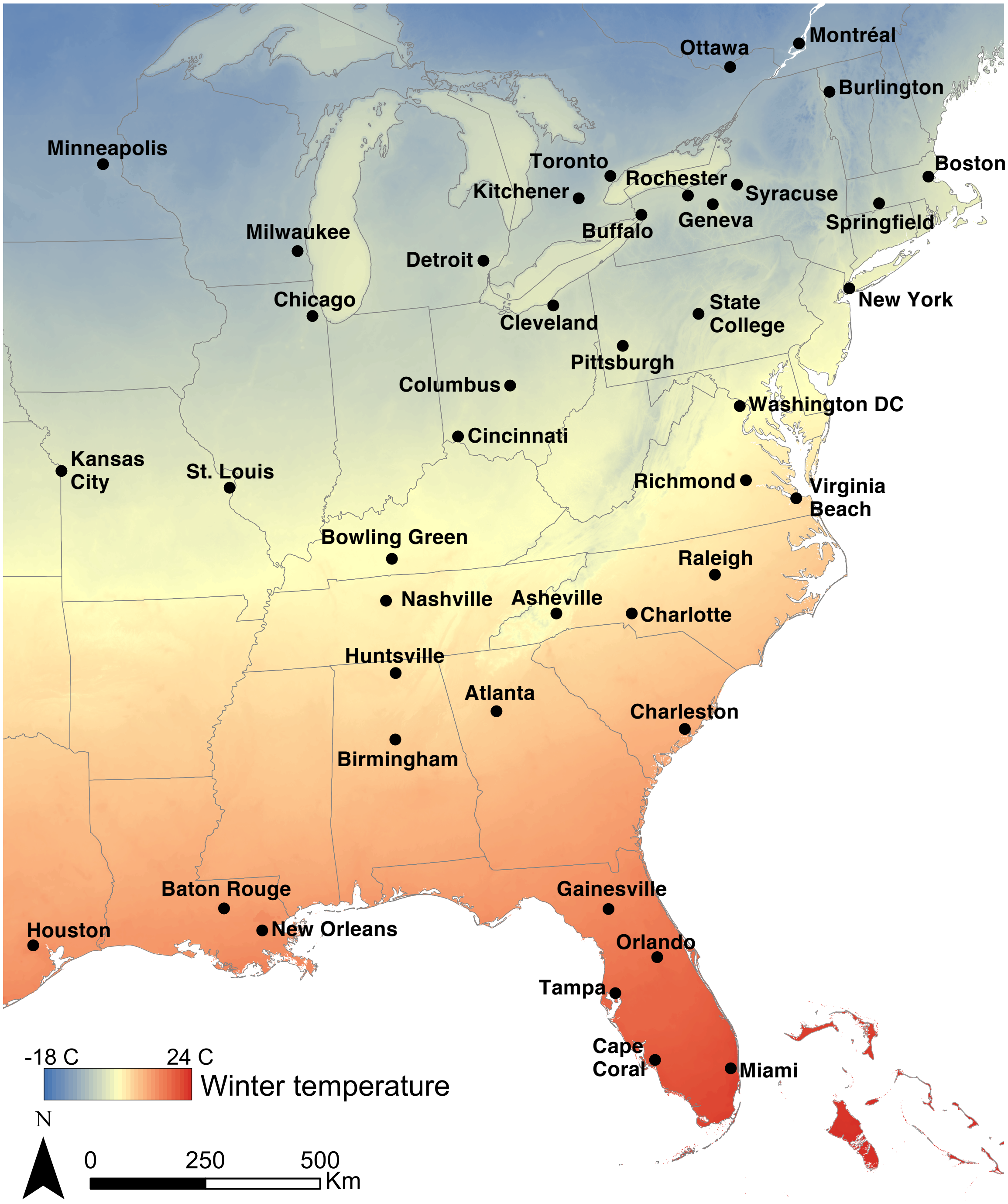

Supplementary Figure 1. Map of 43 cities in North America included in analyses of the distribution of coat color morphs of eastern gray squirrels (*Sciurus carolinensis*). Map was created in ArcMap 10.5.1.

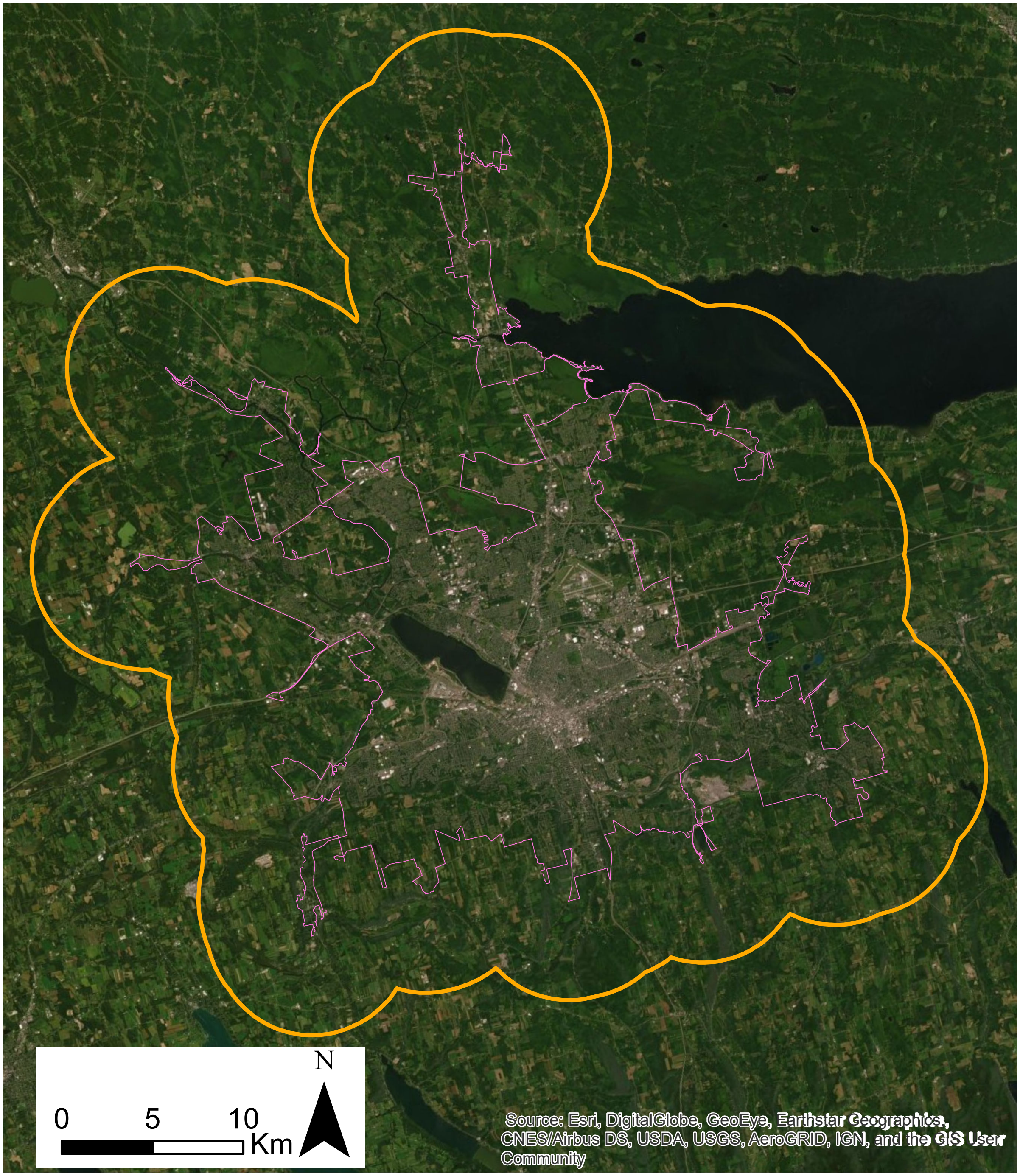

Supplementary Figure 2. Map of Syracuse, NY highlighting methodology for defining the spatial extent of the city footprint (purple line) and buffer (orange line). Map was created in ArcMap 10.5.1 with World Imagery Basemap.

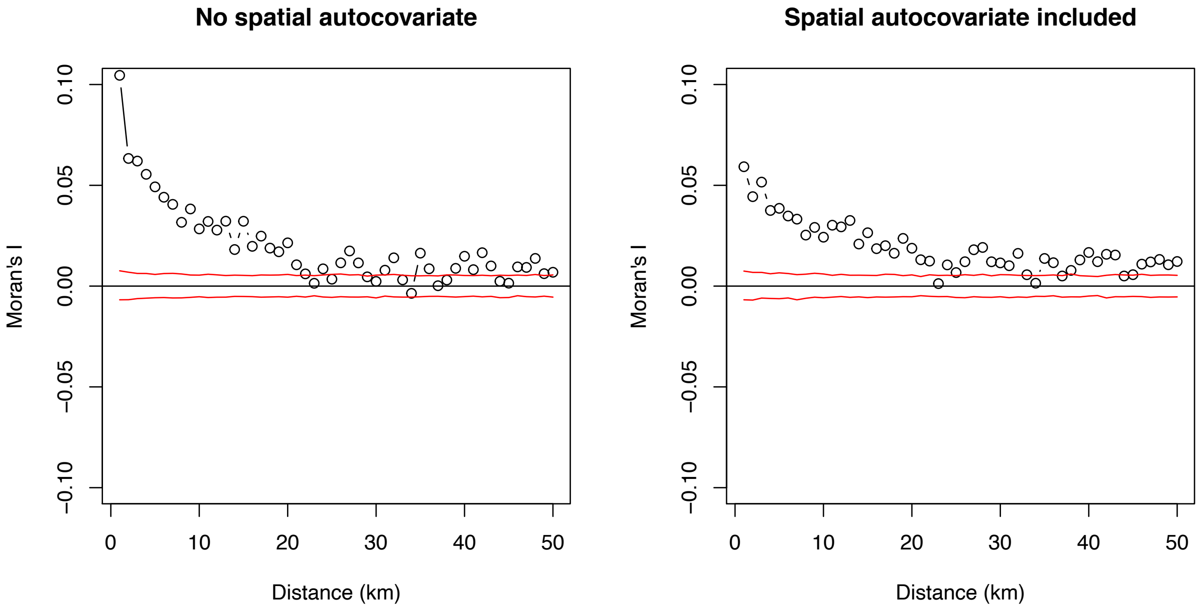

Supplementary Figure 3. Spatial correlograms of residuals from the linear mixed model of the probability of melanism (Supplementary Table 2). Moran's *I* is shown for distance classes of 1 km for a model either without or with a spatial autocovariate included in the model. Red lines show bounds of Moran's *I* beyond which the null hypothesis of $r = 0$ is rejected at a significance value of 0.05, based on Monte Carlo permutations with 999 permutations at each distance class. The bounds of statistical significance are conservative because the analysis does not account for the nesting of observations within cities.
